# X-linked adrenoleukodystrophy: very long-chain fatty acid metabolism is severely impaired in monocytes but not in lymphocytes

**DOI:** 10.1093/hmg/ddt645

**Published:** 2013-12-20

**Authors:** Franziska D. Weber, Christoph Wiesinger, Sonja Forss-Petter, Günther Regelsberger, Angelika Einwich, Willi H.A. Weber, Wolfgang Köhler, Hannes Stockinger, Johannes Berger

**Affiliations:** 1 Center for Brain Research, Medical University of Vienna, Spitalgasse 4, Vienna A-1090, Austria; 2 Institute of Neurology, Medical University of Vienna, Währinger Gürtel 18-20, Vienna A-1090, Austria; 3 Department of Neurology, Fachkrankenhaus Hubertusburg, Wermsdorf D-04779, Germany; 4 Institute for Hygiene and Applied Immunology, Center for Pathophysiology, Infectiology and Immunology, Medical University of Vienna, Vienna A-1090, Austria

## Abstract

X-linked adrenoleukodystrophy (X-ALD) is a fatal neurodegenerative disease caused by mutations in the *ABCD1* gene, encoding a member of the peroxisomal ABC transporter family. The ABCD1 protein transports CoA-activated very long-chain fatty acids (VLCFAs) into peroxisomes for degradation via β-oxidation. In the severest form, X-ALD patients suffer from inflammatory demyelination of the brain. As the extent of the metabolic defect in the main immune cells is unknown, we explored their phenotypes concerning mRNA expression pattern of the three peroxisomal ABC transporters, VLCFA accumulation and peroxisomal β-oxidation. In controls, ABCD1 expression was high in monocytes, intermediate in B cells and low in T cells; ABCD2 expression was extremely low in monocytes, intermediate in B cells and highest in T cells; ABCD3 mRNA was equally distributed. In X-ALD patients, the expression patterns remained unaltered; accordingly, monocytes, which lack compensatory VLCFA transport by ABCD2, displayed the severest biochemical phenotype with a 6-fold accumulation of C26:0 and a striking 70% reduction in peroxisomal β-oxidation activity. In contrast, VLCFA metabolism was close to control values in B cells and T cells, supporting the hypothesis that sufficient ABCD2 is present to compensate for ABCD1 deficiency. Thus, the vulnerability of the main immune cell types is highly variable in X-ALD. Based on these results, we propose that in X-ALD the halt of inflammation after allogeneic hematopoietic stem cell transplantation relies particularly on the replacement of the monocyte lineage. Additionally, these findings support the concept that *ABCD2* is a target for pharmacological induction as an alternative therapeutic strategy.

## INTRODUCTION

X-linked adrenoleukodystrophy (X-ALD; Phenotype MIM number #300100), the most frequent monogenetically inherited peroxisomal disease, causes demyelinating and neurodegenerative processes in the nervous system (1–3). The broad clinical presentation can be grouped into two major phenotypes, adrenomyeloneuropathy (AMN) and inflammatory cerebral adrenoleukodystrophy (CALD). AMN (mean age-of-onset 28 years) is thought to be the default manifestation of X-ALD, a slowly progressive non-inflammatory axonopathy involving the spinal cord and peripheral nerves. This results in a typical triad of spastic paraplegia, sensory involvement and bladder dysfunction (1). In CALD, rapidly progressive, inflammatory cerebral demyelination occurs independently of AMN. Being the most severe form of X-ALD, patients suffer from rapid cognitive and neurological decline and within few years proceed to a vegetative state. For all male X-ALD patients, there is a 60% risk to develop CALD. Most commonly, CALD occurs during childhood before the onset of AMN (35–40%; mean age-of-onset 7 years) and less frequently (20%) in adolescent or adult AMN patients (3,4). About 66% of male X-ALD patients have primary adrenocortical insufficiency (Addison disease). Heterozygous females often develop a milder form of AMN, but rarely adrenal insufficiency (<5%) or the devastating brain inflammation (1,3). If symptoms exceed those of AMN, other explanations than single ABCD1 mutations must be considered (5).

X-ALD is caused by mutations in the *ABCD1* gene, which encodes the ABCD1 protein (formerly adrenoleukodystrophy protein, ALDP), constituting a half ATP-binding cassette (ABC)-transporter in the peroxisomal membrane (6). There is no general genotype–phenotype correlation determining the severity of the disease (2). ABCD1 transports CoA-activated, saturated very long-chain fatty acids (VLCFAs; carbon chain length ≥22 C atoms) from the cytoplasm into peroxisomes for degradation by β-oxidation (7,8). Thus, in ABCD1 deficiency, very long-chain fatty acyl-CoAs are enriched in the cytosol. They can be further elongated by enzymes of the ELOVL (elongation of VLCFAs) family and may be incorporated into different lipids such as phosphatidylcholine, gangliosides or sulphatides and lipoproteins (9). This pathognomonic accumulation of VLCFA in cells and body fluids is used as a diagnostic criterion for X-ALD (2).

The molecular mechanisms underlying the different forms of X-ALD (AMN and CALD) are fundamentally different from each other (10). Moreover, even within different phases during development of the cerebral inflammation and the ineffectiveness of anti-inflammatory therapies, different processes and cell types seem to be involved (10). The systemic circulating immune cells may be crucial for the phenotype. At an early stage of brain inflammation, allogeneic hematopoietic stem cell transplantation (HSCT) can be applied. This is currently the only curative treatment option for CALD (11). Beginning demyelination and inflammation can be detected in brain magnetic resonance imaging (MRI), preceding the first symptoms of CALD (12). In addition, decreased magnetic resonance perfusion imaging appears to be an early predictor of lesion progression in CALD (13). Therefore, MRI plays an important role in the monitoring of patients. The ‘time window’ for finding an appropriate donor is narrow, because the disease progresses rapidly at that stage; and it takes at least 6 months after HSCT for the inflammation to halt (14). Moreover, for some patients no compatible donor is available. In order to circumvent this limitation, Cartier and colleagues developed a protocol for autologous HSCT. They corrected the patients' own CD34^+^ stem cells *ex vivo* with a lentiviral vector encoding intact ABCD1 protein. Using this strategy, they cured the cerebral inflammation of two childhood CALD patients (15). In spite of this success, currently there is no treatment available for CALD patients with advanced inflammation at diagnosis, for AMN patients, and for symptomatic heterozygous females. This leaves the majority of X-ALD patients without effective treatment options. Thus, there is still an urgent need to develop novel therapeutic strategies for X-ALD (11).

One principle for a novel therapeutic approach is the induction of the homologous *ABCD2* gene encoding ABCD2 protein (formerly adrenoleukodystrophy related protein, ALDRP), also localized in the peroxisomal membrane. Functional overlap has been demonstrated *in vitro* in X-ALD fibroblasts (16) and *in vivo* in a mouse model of X-ALD (17), where overexpression of ABCD2 could compensate for ABCD1 deficiency. Most likely, due to differing expression levels of the two genes in different cell types and tissues, endogenous ABCD2 cannot replace ABCD1 functionally (18,19).

Our objective for the present study was to determine whether the main immune cell types are equally affected in X-ALD. Therefore, we hypothesized that the expression level of *ABCD2* is a critical determinant for manifestation of the pathological phenotype in the different immune cell types of X-ALD patients. Here, we show that the three peroxisomal ABCD transporters (ABCD1, ABCD2 and ABCD3) are differentially expressed in the various immune cell types. The expression profiles support the concept of ABCD2 as a crucial modulator of the severity of the defect in VLCFA degradation in immune cells in X-ALD.

## RESULTS

### ABCD1 and ABCD2 are differentially expressed in CD34^+^-derived immune cells

**Figure 1.**
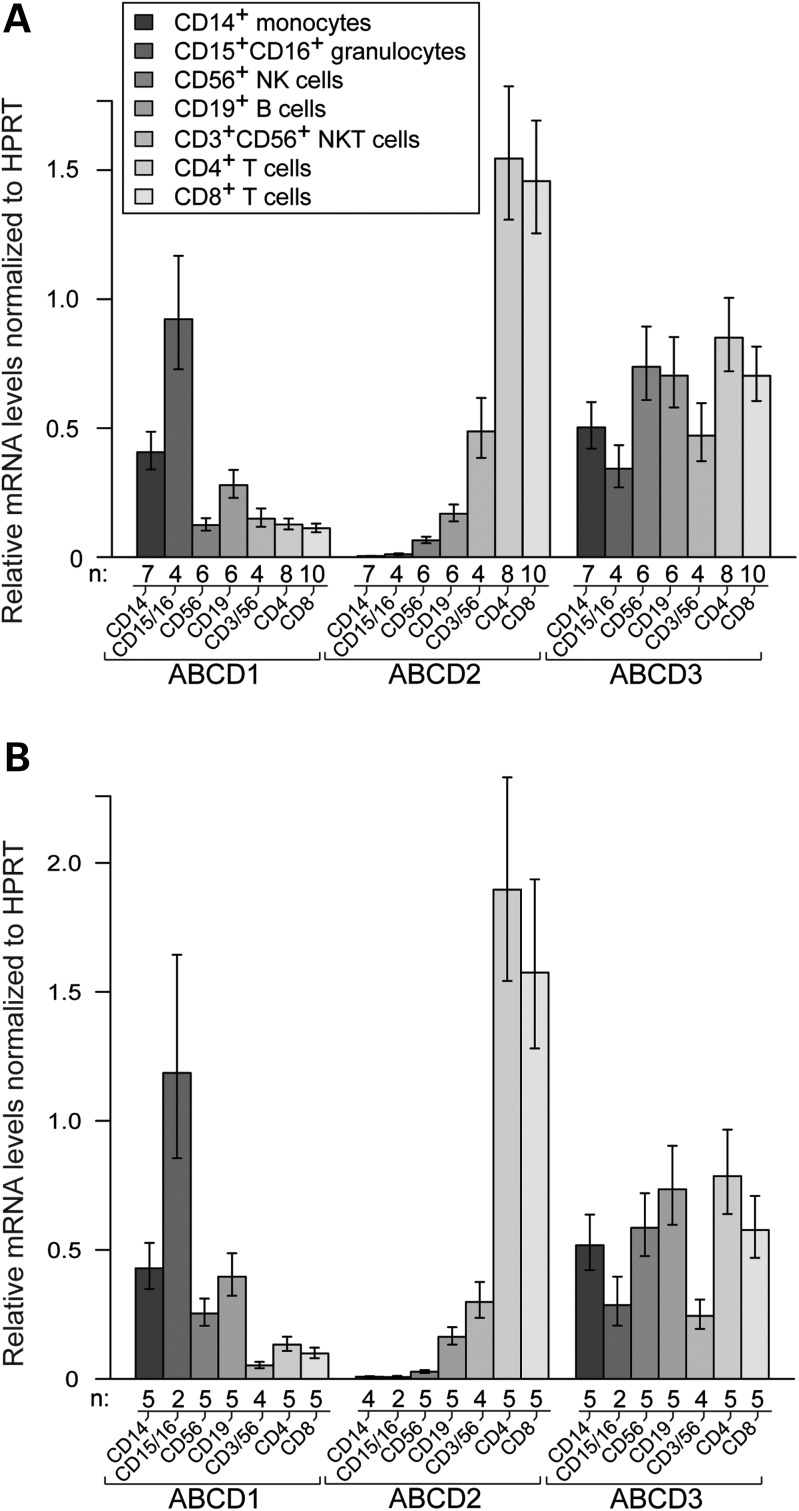
Peroxisomal ABCD transporters are differentially expressed in the main immune cells. The mRNA levels of ABCD1, ABCD2 and ABCD3 were measured by qRT-PCR in the indicated immune cell types in (**A**) healthy controls and (**B**) AMN patients. Absolute copy numbers of mRNA were normalized to the reference gene hypoxanthine phosphoribosyltransferase (HPRT). Values represent means ± SEM. The number of individuals (*n*) is indicated below the graphs.

In order to explore the biological relevance of ABCD1 in the major CD34^+^-derived immune cell types, we compared the basal ABCD1 mRNA levels with those of the other peroxisomal ABC transporters, ABCD2 and ABCD3 in healthy male adults (25–48 years of age) by using quantitative reverse transcription (qRT)-coupled PCR. Venous blood was collected in the morning under fasting conditions. Purity of isolated cell types was typically ≥95% as determined by flow cytometry analysis (Supplementary Material, Fig. S1). The gene-specific differences between the cell types were quite distinct and consistent. In general, there was a surprisingly low inter-individual variability in the mRNA expression profile of the three genes (Fig. 1A; cf. raw data in Supplementary Material, Fig. S2). The results are depicted as a ratio to HPRT mRNA levels, which shows minor variability across different cell types (Supplementary Material, Fig. S2). Among the analysed cell types, ABCD1 mRNA levels were the highest in neutrophil granulocytes (CD15^+^/CD16^+^) followed by monocytes (CD14^+^). For qRT-PCR, we separated neutrophils from the minor eosinophil granulocyte fraction (CD15^+^/CD16^−^), as neutrophils are the most important granulocyte population during inflammation. However, eosinophils are transcriptionally more active than neutrophils (20). ABCD1 mRNA levels were intermediate in B cells (CD19^+^) and lowest in natural killer (NK) cells (CD3^−^/CD56^+^), NKT cells (CD3^+^/CD56^+^) and T cells (CD4^+^ and CD8^+^). In contrast to the relatively high mRNA levels of ABCD1 in monocytes and granulocytes, the ABCD2 mRNA was barely detectable in these cells (Fig. 1A). In B cells and NK cells, the mRNA levels of ABCD1 and ABCD2 were comparable. However, in T cells (CD4^+^ and CD8^+^), we found the highest ABCD2 mRNA levels, exceeding those of ABCD1 by an order of magnitude (Fig. 1A). The ABCD3 mRNA was rather equally distributed in all investigated immune cells, with slightly lower levels in neutrophil granulocytes and NKT cells (Fig. 1A). This is also in good agreement to previous studies showing that the *ABCD3* gene is ubiquitously expressed (21).

In order to avoid influences resulting from a biased selection of a single housekeeping gene (HPRT), we investigated the ABCD 1–3 expression levels normalized to an additional housekeeping gene (GAPDH). The results depicted as relative mRNA levels based on the geometric means of HPRT and GAPDH, show a similar profile (Supplementary Material, Fig. S3).

### No compensatory regulation of the three peroxisomal ABC transporters in immune cells of AMN patients

**Figure 2.**
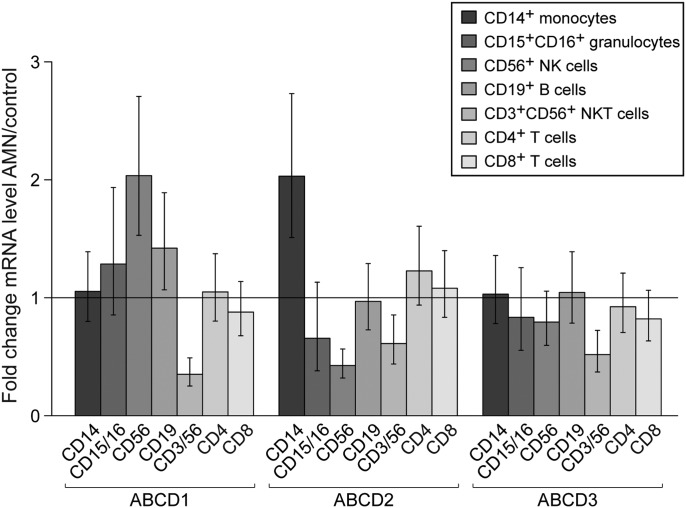
There is neither compensatory feedback regulation of ABCD1 nor up-regulation of ABCD2 or ABCD3 mRNAs in immune cells of AMN patients. Statistical analysis of the fold change of ABCD1, ABCD2 and ABCD3 mRNA levels normalized to HPRT for AMN versus healthy control revealed no statistically significant differences. ABCD2 mRNA levels in monocytes and granulocytes were close to the detection limit in both, controls and AMN patients, therefore apparent differences in their expression levels are not considered relevant. *F*-test showed no significant difference for the population factor (AMN; healthy controls) in all investigated cell types with the exception of NKT cells. However, further analysis of NKT cells with Student's *t*-test did not confirm a significant difference.

Next, we analysed the basal mRNA levels of the peroxisomal ABC transporters in the immune cell types of X-ALD patients in order to investigate potential compensatory regulation of gene expression. We recruited adult AMN patients (29–43 years of age) with a pure non-inflammatory AMN phenotype and without Lorenzo's Oil treatment. We focused on this group of patients in order to determine the intrinsic cell properties in ABCD1 deficiency, without confounding secondary alterations due to inflammatory responses. We expect the results obtained in AMN to be representative for all forms of X-ALD in males, as the extent of VLCFA accumulation does not differ between CALD and AMN patients when comparing plasma or fibroblasts (1). The same cell populations as for the healthy controls were isolated from five AMN patients, which all had normal white blood cell counts at the time of analysis (Supplementary Material, Table S1). The mRNA levels of ABCD1, ABCD2, and ABCD3 in the patient collective (Fig. 1B) showed a similar distribution as in healthy controls (Fig. 1A). None of the AMN patients carried a mutation affecting the transcription of the *ABCD1* gene. There was no statistically significant compensatory induction of ABCD1, ABCD2 and ABCD3 in any cell type of AMN patients (Fig. 2). Taking into account that upon overexpression ABCD2 can compensate for ABCD1 deficiency, these results imply that in AMN monocytes and granulocytes should be most severely affected.

### The extent of VLCFA accumulation in AMN differs widely between the immune cell types

**Figure 3.**
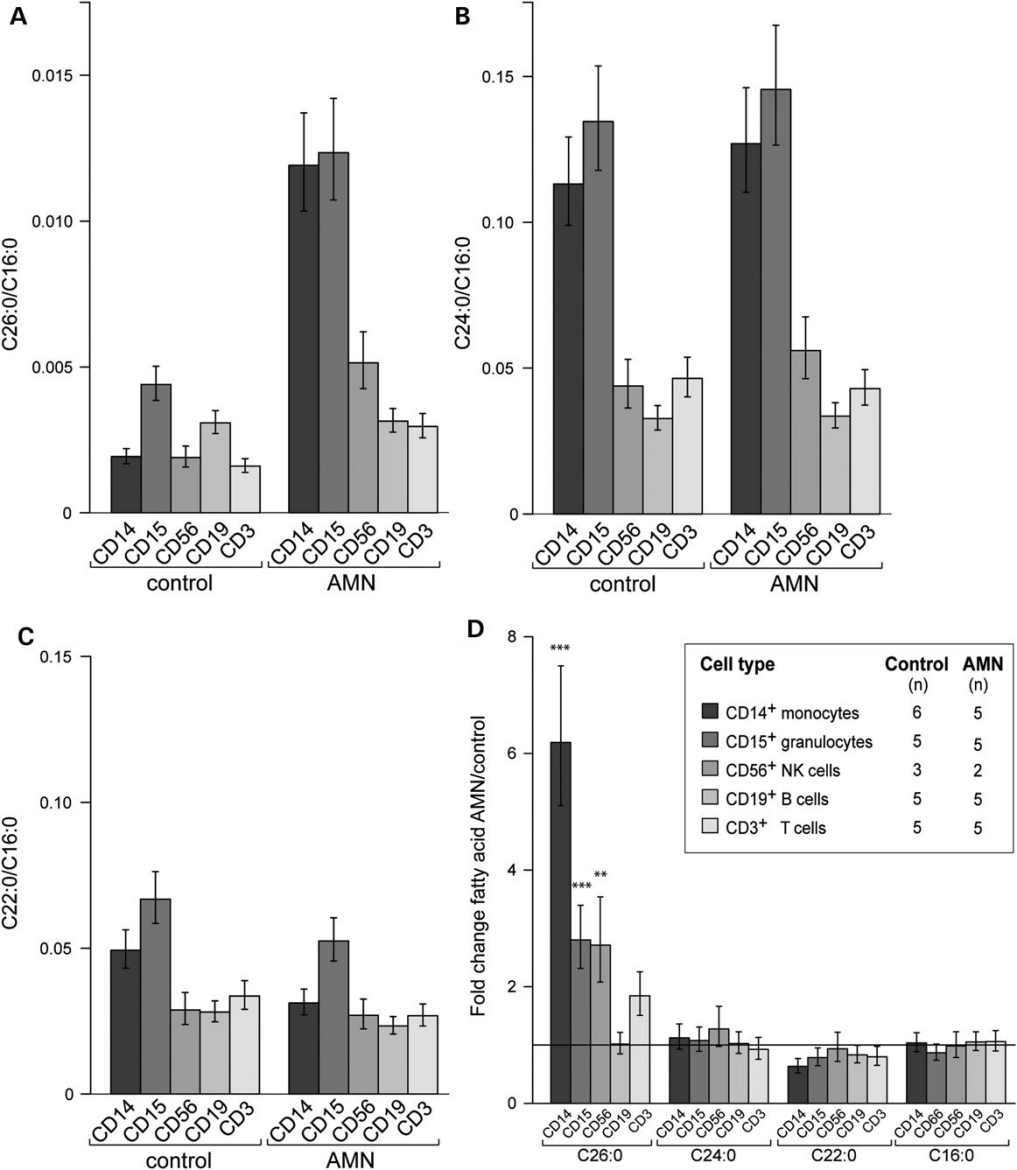
Fatty acid levels in immune cells of healthy controls and AMN patients. The concentrations of C26:0, C24:0, C22:0 and C16:0 were determined by GC–MS in the indicated immune cell types. The relative amounts of (**A**) C26:0, (**B**) C24:0, (**C**) C22:0 normalized to C16:0 are shown. The statistical analysis of the accumulation of VLCFA is described in (**D**) as fold change of FA levels for AMN versus healthy control. Values represent means ± SEM. For all panels, the number of individuals (*n*) is indicated in the inset of (D). Note the different scaling (Student's *t*-test, ***P < 0.001; **P < 0.01).

All tissues, body fluids and cell types that have been investigated from X-ALD patients accumulate saturated VLCFA, albeit to variable degrees (1). Therefore, we asked to what extent the different immune cell types isolated from blood of AMN patients accumulate VLCFA. We measured the total amount of the fatty acids (FAs) C26:0, C24:0, C22:0 and, for normalization, C16:0 by using gas chromatography–mass spectrometry (GC–MS) in the following isolated cell types: monocytes (CD14^+^), granulocytes (CD15^+^), B cells (CD19^+^), NK cells (CD3^−^/CD56^+^) and T cells (CD3^+^) of controls and AMN patients (Fig. 3). The relative amount of each VLCFA is displayed as a ratio to C16:0, a long-chain FA that does not differ between healthy controls and AMN patients (Fig. 3D; Supplementary Material, Fig. S5D). In healthy controls the absolute amounts of C26:0 were approximately 1/20-1/40 of C24:0 and 1/20 of C22:0 (Supplementary Material, Fig. S4 and S5). Among the cell populations isolated from healthy controls, granulocytes (CD15^+^) and B cells (CD19^+^) displayed the highest levels of C26:0, whereas monocytes, NK cells and T cells contained about half as much (Fig. 3A, left panel; Supplementary Material, Fig. S5A, left panel). Monocytes and granulocytes showed higher levels of C24:0 than NK cells, B cells and T cells (Fig. 3B, left panel; Supplementary Material, Fig. S5B, left panel).

When comparing the cell type-specific levels of C26:0/C16:0 of the AMN patients and healthy controls, we found a statistically significant 6-fold increase in monocytes and a 3-fold increase in granulocytes and NK cells. In contrast, B cells and T cells showed 2-fold accumulation of C26:0 compared with the control group (Fig. 3A, right panel and 3D). Similar results were obtained when considering the absolute amounts (Supplementary Material, Fig. S5A) or the ratio C26:0/C22:0 (Supplementary Material, Fig. S5E). Thus, cell types displaying high levels of ABCD1 mRNA and low or no detectable expression of ABCD2 showed the highest degree of C26:0 accumulation in AMN patients.

We observed no statistically significant differences in the C24:0 levels between AMN patients and healthy controls (Fig. 3B and D). However, in all cell types of the AMN group there was a trend towards decreased C22:0 levels, which was most pronounced in monocytes (*P*-value 0.08) (Fig. 3C and D).

For diagnostic purposes in X-ALD patients frequently leukocytes are used. Diagnostic leukocyte preparations contain ∼50–70% granulocytes, 3–10% monocytes and 20–25% lymphocytes. As the cell types with the highest C26:0 levels are the most abundant in such preparations, the total C26:0 accumulation was reliably established (at least 5-fold) in our patient collective (data not shown) and (22).

### β-Oxidation activity reflects the extent of VLCFA accumulation in immune cells from AMN patients

The accumulated VLCFA can be derived from external sources or from endogenous FA chain elongation. Thus, the accumulation does not necessarily reflect the degradation of VLCFA. Therefore, we investigated the rate of peroxisomal β-oxidation. Because of the limited biological material and the relatively high amounts needed, the β-oxidation measurements were restricted to the most abundant immune cells, namely monocytes, B cells and T cells.

**Figure 4.**
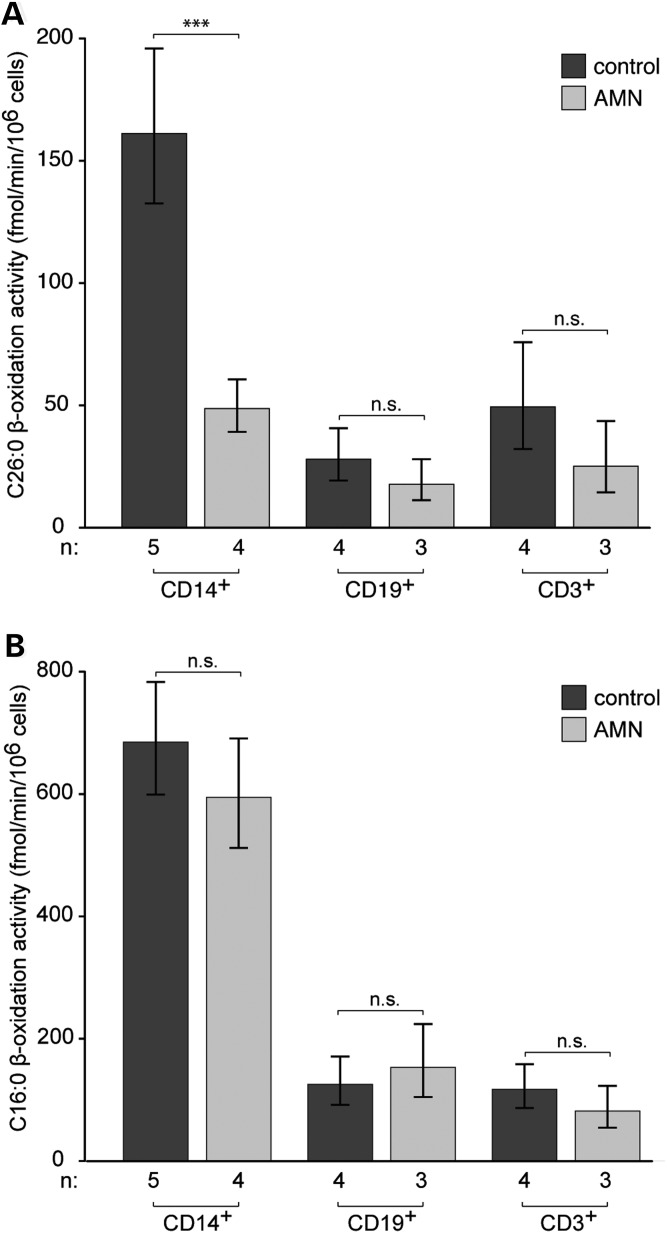
β-Oxidation in immune cells of healthy controls and AMN patients. The activity of (**A**) peroxisomal C26:0 and (**B**) mitochondrial C16:0 β-oxidation were measured in monocytes (CD14^+^), B cells (CD19^+^) and T cells (CD3^+^). The rate of β-oxidation is expressed as fmol ^14^C-labeled acetate released/min/10^6^ cells. Values represent means ± SEM. The number of individuals (*n*) is indicated below the graphs (Student's *t*-test, ***P < 0.001; n.s., not significant).

In healthy controls, the peroxisomal (substrate C26:0) and mitochondrial (substrate C16:0) β-oxidation activity of monocytes was ∼3- to 4-fold higher than those rates of B and T cells (Fig. 4; cf. raw data Supplementary Material, Fig. S6). The metabolism of C16:0 was not affected in any of the analysed cell types of AMN patients (Fig. 4B). However, we found a highly significant decrease of 69.7% in the C26:0 β-oxidation activity of AMN monocytes compared with the healthy controls. In contrast, in AMN T cells, there was a trend toward a decrease in the C26:0 β-oxidation activity that did not reach statistical significance. In AMN B cells, the β-oxidation activity was approaching control values (Fig. 4A).

Taken together, these results provide further evidence that in X-ALD, VLCFA metabolism is differentially affected in the various immune cell types, ranging from severe in monocytes to moderate in T cells and no impairment in B cells.

## DISCUSSION

To date, transplantation of allogeneic hematopoietic stem cells or autologous hematopoietic CD34^+^ stem cells corrected by gene therapy have been the only effective treatments for CALD (14). However, the mechanisms behind the benefit of these therapies are poorly understood. Here, we attempt to shed light on the pathophysiology of the CD34^+^-derived cell types, as one or more of these must play a crucial role in HSCT. In addition, we identified potential target cells for future therapeutic strategies, such as pharmacological upregulation of the redundant *ABCD2* gene to ameliorate the metabolic impairment and possibly halt the inflammation in CALD.

For the first time, the expression profiles of the peroxisomal ABC transporters and the biochemical phenotype were directly compared in isolated CD34^+^-derived immune cell types of AMN patients and healthy controls. We found that the severity of the impairment in VLCFA metabolism differs strongly between these cell types and correlates with the expression levels of the peroxisomal ABC transporters ABCD1 and ABCD2.

We identified two cell types with a robust pathognomonic phenotype, monocytes and granulocytes, which in healthy controls have a high expression of ABCD1 and no or barely detectable expression of ABCD2 mRNA in common. In these cells, we observed the highest accumulation of VLCFA in X-ALD, and in monocytes peroxisomal β-oxidation activity for C26:0 was significantly reduced (<30% of control values). Interestingly, these two cell types are functionally related and also build a cluster on the basis of gene expression (23). Our findings that monocytes are severely affected by the disease are in line with several other publications. Increased cytokine levels, especially tumor necrosis factor (TNF)-α, were previously detected in peripheral blood mononuclear cells (PBMCs) from X-ALD patients stimulated by lipopolysaccharide (LPS) (24); additionally, elevated levels of interleukin (IL)-2 and IL-1α were found (25). Most likely, this TNF-α production came from peripheral monocytes since stimulation by phytohaemagglutinin, a T cell activator, did not increase TNF-α amounts (24). Also in a mouse model of X-ALD, Yanagiasawa and coworkers showed that peritoneal macrophages from *Abcd1*-deficient mice secreted significantly elevated levels of TNF-α, IL-6 and IL-12p70 upon stimulation by LPS and interferon-γ (IFN-γ) compared with wild-type mice (26). Chitotriosidase activity, an activation marker for macrophages, was found to be elevated in plasma and cerebral spinal fluid (CSF) of CALD patients (27). Recent findings also showed elevated proinflammatory chemokines (IL-8, IL-1ra, monocyte chemotactic protein (MCP)-1, macrophage inflammatory protein (MIP)-1β) that induce migration of peripheral immune cells to the site of inflammation in CSF of CALD patients. Interestingly, IL-8 and MCP-1 levels correlated significantly with MRI severity, whereby IL-8 is speculated to mediate blood–brain barrier breakdown, and MCP-1 is a chemotaxin for monocytes (28). Taken together, these observations not only establish a major role for monocytes in the pathology of CALD but also provide evidence for functional abnormalities.

Here, we found that monocytes have a severe intrinsic metabolic defect in X-ALD. We suggest that this is responsible for an altered cytokine production leading to further changes in the immune response, for example T cell activation in a predominant Th1 response (25). Our results show that T cells of X-ALD patients express high levels of ABCD2 mRNA and hardly accumulate C26:0 (<2-fold increase) compared with controls. Thus, in X-ALD, primary T cells, in contrast to monocytes, apparently have only a subtle defect in their VLCFA metabolism.

Strikingly, B cells, which express intermediate levels of ABCD2 mRNA, are barely affected metabolically by *ABCD1* deficiency; VLCFA do not accumulate and the β-oxidation activity for C26:0 is similar to that of controls. These results differ from published reports of 3- to 7-fold accumulation of C26:0 in a human X-ALD B-cell line (29,30). However, there are major differences in experimental settings that probably accounts for this discrepancy. Whereas we analysed freshly isolated primary B cells without any further handling or cultivation steps, Uto and colleagues (29) used lymphoblast cell lines in long-term cultures containing 10% fetal bovine serum, which has been shown to alter the expression levels of the peroxisomal ABC transporters (31,32).

Taken together, we observed that in cells with minimal or no compensatory ABCD2 (monocytes and granulocytes), the VLCFA accumulation was the most severe (3- to 6-fold) in X-ALD. However, when ABCD2 is expressed at moderate levels, as for example in B cells and NK cells, or at high levels, even exceeding that of ABCD3 mRNA, as in T cells, the extent of accumulation of C26:0 in X-ALD cases was <2-fold. Thus, in *ABCD1* deficiency there is an inverse relationship between the level of ABCD2 and the extent of the metabolic impairment.

The trigger(s) for the cerebral inflammation in X-ALD are not known. Spontaneous myelin breakdown, due to membrane destabilization caused by the incorporation of abnormal VLCFA into myelin-specific lipids has been suggested as a cause for the initiation of inflammation (33). Alternatively, traumatic brain injury could act as an extrinsic factor, promoting an opening of the blood–brain barrier initiating inflammation and demyelination (34). In both cases, it is plausible that glycolipid-restricted T cells recognize their antigen within the brain. Probably the antigen is presented by CD1 molecules on perivascular cells in an MHC class I independent manner leading to a further recruitment of proinflammatory cells (35). As shown by Ito and co-workers, CD8^+^ T cells invade the brain at an early stage of inflammation and cause cytolysis of oligodendrocytes (35). CD8^+^ T cells seem to play a crucial role in the initiation and progression of the inflammation, but as shown in the present study, have no severe intrinsic metabolic defect. Thus, in X-ALD, T cells are able to execute their immune functions. However, their response might be altered due to changed cytokine production by macrophages.

Monocytes, the progenitor cells of macrophages, and granulocytes are key players in inflammatory processes. Further differentiation of monocytes into macrophages with recombinant human macrophage colony-stimulating factor did not change the ABCD2 expression status (remained below the level defined as being expressed) (36). Thus, we postulate that in CALD, the invading activated macrophages have the same metabolic defect as their CD14^+^ precursor monocytes investigated in our study. In order to handle the increasing FA uptake from phagocytosed myelin debris - in the absence of appropriate capacity for degradation of VLCFA - these are esterified to cholesterol, as observed by lipid analysis of autopsy material from affected lesions (37). Intracellular VLCFA-cholesterol esters deposited inside macrophages have been visualized as crystalline structures (38) and are expected to promote cellular stress. It has been demonstrated that the immunoreactivity of oxidative stress markers correlates with the degree of inflammation and myelin breakdown (39). Moreover, Eichler and co-workers showed that VLCFA-enriched lipids, in particular the lysophosphatidylcholine fraction, can lead to microglia activation and apoptosis (40). Thus, next to brain derived cell types the intrinsic molecular defect in circulating monocytes/macrophages seems to be involved in progression of inflammation.

The metabolic defect in macrophages may also provide an explanation for the failure of anti-inflammatory treatment attempts for CALD, as they do not target the intrinsic defect of macrophages. Thus, it is tempting to speculate that correction of the transport of VLCFA for peroxisomal β-oxidation in invading macrophages could halt brain inflammation in CALD. The lack of generalized genotype–phenotype correlation in X-ALD is obvious from the fact that the same familial mutations in *ABCD1* can give rise to all different phenotypes (10). No mutations have been found in the *ABCD2* gene of X-ALD patients and genetic association studies indicated that *ABCD2* is unlikely to be a genetic modifier locus (41). However, as ABCD2 is barely expressed in some cell types including the monocytic lineage, the level of expression in particular cell types could still be a crucial determinant of the phenotype. Therefore, one possible way to restore the metabolic status of macrophages could be mediated by an induction of ABCD2 in this cell type (11). A previous clinical trial investigated the ability of the histone deacetylase inhibitor valproic acid to normalize VLCFA accumulation in X-ALD patients via an up-regulation of ABCD2 (42). However, the results were inconclusive because PBMCs were analysed and they represent a mixed cell population of mostly T cells (60–75%) and only 10–30% monocytes.

In conclusion, we provide evidence that it is crucial to analyse distinct cell types to evaluate the success of treatment at the level of gene expression or biochemical parameters. Our results show that monocytes rather than lymphocytes are the most severely affected immune cell population that needs to be targeted by therapeutic intervention in X-ALD. Furthermore, our findings provide a molecular basis for explaining why allogeneic and autologous HSCT are effective at arresting the cerebral inflammation, namely by the replacement and the functional metabolic restoration of the monocyte lineage.

## MATERIALS AND METHODS

### Patients and healthy volunteers

In the study included were five AMN patients and 10 healthy volunteers, all male and of Caucasian origin (age 25–48 years). Patients with ‘pure’ AMN had isolated spinal cord disease with or without adrenal insufficiency but without cerebral demyelinating lesions at brain MRI. White blood cell counts did not reveal any signs of inflammation (Supplementary Material, Table S1). A total volume of 300 ml blood was collected with informed consent in accordance to the Declaration of Helsinki including all actual revisions. The study was approved by the Ethical Committee of the Medical University of Vienna.

### Pre-enrichment of PBMCs and granulocytes

Blood was collected by venipuncture under fasting conditions into heparin tubes and diluted 1:1 with phosphate-buffered saline (PBS) at room temperature. To separate the PBMCs from other blood components, the diluted blood was overlaid onto tubes containing Pancoll separating medium (density of 1.077 g/ml; PAN-Biotech, Aidenbach, Germany) and centrifuged for 25 min at 500*g* at room temperature without brake. For granulocyte isolation, a Percoll (density of 1.131 g/ml; Sigma-Aldrich, St. Louis, MO, USA) step gradient was used with 72% (v/v) Percoll in PBS at the bottom and 63% (v/v) Percoll in PBS on top. Diluted blood was overlaid and centrifuged for 25 min at 500*g* at room temperature without brake. Afterwards, the PBMCs and granulocyte fraction (mainly neutrophils and ∼5–10% eosinophils) were removed and washed with PBS.

### Magnetic activated cell sorting

The following microbead-coupled antibodies were used for isolation of specific cell types from PBMCs: CD3^+^, CD4^+^, CD8^+^, CD14^+^, CD19^+^, CD56^+^ and an NKT cell separation kit (Miltenyi Biotec, Bergisch Gladbach, Germany; Supplementary Material, Table S2). For the measurement of the ABC transporter mRNAs in neutrophils, a CD16^+^ microbead-coupled antibody (Miltenyi Biotec) was used to further separate them from eosinphils in the granulocyte fraction. Specific cell types were separated by either positive or negative selection according to the instructions of the manufacturer. In some cases, the unlabelled cell fraction (flow-through after positive selection) was recovered and used for the isolation of an additional cell type. Purity of the cells was measured on a BD FACS Calibur flow cytometer (Becton Dickinson, NJ, USA) and analysed using FlowJo software (Treestar Inc., Ashland, OR, USA).

### Quantitative real-time PCR analysis

Purified cell types were lysed in Trizol following the manufacturer's (Invitrogen, Paisley, UK) instructions using 1 ml Trizol reagent per 10^6^ cells. For homogenization, the lysate was passed through a QIAshredder spin column (Qiagen, Bothell, WA, USA). The isolated total RNA fraction was purified further using RNeasy Mini Kit and RNase-free DNase digestion (Qiagen). RNA concentration was measured based on optical density using a Nanodrop spectrophotometer (Peqlab, Erlangen, Germany). Total RNA (80 ng) was reverse transcribed using the iScript™ cDNA Synthesis Kit (Bio-Rad, Hercules, CA, USA). The cDNA was diluted 1:5 in RNase-free water and aliquots corresponding to 4 ng of total RNA were amplified in technical duplicates by qRT-PCR. ABCD1, ABCD3 and HPRT were detected by the SYBR Green method using SsoFast™ EvaGreen Mix (Bio-Rad). ABCD2 was detected by the Taqman method using SsoFast™ Probes Supermix (Bio-Rad). Both methods were carried out using the CFX96 Realtime System (Bio-Rad) according to the manufacturer's instructions. Thermocycler settings are available as supplementary information (Supplementary Material, Table S3).

For amplification and detection of ABCD1 cDNA, the primers 5′-GAGAACATCCCCATCGTC-3′ (forward, nucleotide position 1828) and 5′-TGTAGAGCACACCACCGTA-3′ (reverse, nucleotide position 1996) were used (GenBank™ Accession No. NM_000033.3). For ABCD2 cDNA, the primers 5′-TCCTACACAATGTCCATCTCT-3′ (forward, nucleotide position 1883) and 5′-AGGACATCTTTCCAGTCCA-3′ (reverse, nucleotide position 1961) as well as the TaqMan fluorescent probe 5′-Cy5-CAAAGAGAAGGAGGATGGGATGC-BHQ2-3′ (nucleotide position 1915) were used (GenBank™ Accession No. AJ000327.1). For ABCD3, the primers 5′-CGGCTCATCACAAACAGTGA-3′ (forward, nucleotide position 811) and 5′-AGGTGTTCCACCAGTTTTCG-3′ (reverse, nucleotide position 908) were used (GenBank™ Accession No. M81182.1). For HPRT, the primers 5′-CCCTGGCGTCGTGATTAGT-3′ (forward, nucleotide position 182) and 5′-CAGGTCAGCAAAGAATTTATAGCC-3′ (reverse, nucleotide position 401) were used (GenBank™ Accession No. NM_000194). For GAPDH, the primers 5′-AGGTCATCCATGACAACTT-3′ (forward, nucleotide position 560) and 5′-AGTCTTCTGGGTGGCAGT-3′ (reverse, nucleotide position 636) as well as the TaqMan fluorescent probe 5′-FAM-CATGACCACAGTCCATGCCA-TAMRA-3′ (nucleotide position 597) were used (GenBank™ Accession No. NM_002046)._Standard curves of known copy numbers were generated for quantification using linearized plasmids containing human ABCD1, ABCD2, ABCD3 and for normalization HPRT and GAPDH cDNA.

### Extraction of FAs and gas chromatography–mass spectrometry analysis

The following FAs were used as standards for quantification: [3,3,5,5-^2^H_4_]-hexacosanoic acid (^2^H_4_-C26:0), [3,3,5,5-^2^H_4_]-docosanoic acid (^2^H_4_-C22:0) and [3,3,5,5-^2^H_4_]-tetracosanoic acid (^2^H_4_-C24:0) were obtained from Dr Herman J. ten Brink from the Free University Hospital, Amsterdam, the Netherlands. [7,7,8,8-^2^H_4_]-Palmitic acid (^2^H_4_-C16:0) was from Cambridge Isotope Laboratories Inc., Andover, MA, USA. Cell pellets were suspended in distilled water and sonicated three times for 30 s on ice. Protein concentration was determined by the method of Lowry using bovine serum albumin as standard (42). Extraction and GC–MS analysis was performed as described (22), using 100 µl internal standard mixture (containing deuterium-labelled free FAs including 0.1 µg/ml ^2^H_4_-C26:0, 0.5 µg/ml ^2^H_4_-C22:0 and ^2^H_4_-C24:0, respectively, and 10 µg/ml ^2^H_4_-C16:0 in toluene) per vial. Data were analysed utilizing calibration curves obtained from the appropriate non-labeled FAs.

### β-Oxidation of 1-^14^C-labeled FAs

[1-^14^C]-palmitic acid (C16:0; ARC 0172A) and [1-^14^C]-hexacosanoic acid (C26:0; ARC 1253) were obtained from American Radiolabeled Chemicals (St. Louis, MO, USA). Free FAs in ethanol were aliquoted into glass reaction tubes, dried under a stream of nitrogen and solubilized in 10 mg/ml α-cyclodextrin by ultrasonication. β-Oxidation of labeled FAs to acetate was carried out essentially as described previously (8,43). In short, the reaction mix of 250 µl contained 4 µm of labeledFA, 2 mg/ml α-cyclodextrin, 30 mm KCl, 8.5 mm ATP, 8.5 mm MgCl_2_, 1 mm NAD^+^, 0.17 mm FAD, 2.5 mm l-carnitine, 0.16 mm CoA, 0.5 mm malate, 0.2 mm EDTA, 1 mm DTT, 250 mm sucrose and 20 mm Tris, pH 8.0. Reactions were started by addition of 1–2 × 10^6^ cells, which had been stored at −80 °C, carried out for 1 h at 37°C and stopped by addition of KOH and heating to 60°C for 1 h. After protein precipitation by HClO_4_, a Folch partition was carried out and ^14^C-acetate was determined in the aqueous phase by scintillation counting.

### Statistical analyses

Linear mixed effect analyses (44–48) were used to describe the relationships between the experimental *responses* (mRNA copy number, FA amount and β-oxidation activity) and three *primary* covariates (type of gene, FA, β-oxidation) in the main immune cell types (cellType) of healthy controls and AMN patients. Replicated recordings were obtained. We modeled the *subject* factor as a random effect and the gene/FA/β-oxidation factor, the *population* (healthy controls; AMN patients) factor and the cell type factor as fixed effects. Separate fixed effect models incorporating an interaction term between primary covariate and the population factor were fitted within the levels of cell type factor. A variance stabilizing log transformation of the response was used for all analyses.

$$\eqalign{&\hbox{Log}\,\,(\hbox{response}) ={\hbox{cellType}} / ({\rm\,primary} \cr&+ \hbox{population} + \hbox{primary}:\hbox{population})|\hbox{subject}.}$$

The interaction term (primary:population) enables different changes (slopes) between the two populations for each level in the primary covariates to be incorporated in the fixed effect model. All terms of the equation are defined in italics in the text.

We tested the significance of terms in the fixed effect model using the conditional *F*-test. A significant fixed effect term was further tested using Student's *t*-test. Multiple testing was taken into account using simultaneously calculated *z*-values for the covariates of interest, obtaining the *P*-values from a multivariate Student's *t*-distribution.

## SUPPLEMENTARY MATERIAL

Supplementary Material is available at *HMG* online.

## FUNDING

This work was supported by the EU project ‘LEUKOTREAT’ (241622), the Austrian Science Fund (FWF) (P26112-B19) and the European Leukodystrophy Association (ELA) Germany. Funding to pay the Open Access publication charges for this article was provided by the Austrian Science Fund (FWF) (P26112–B19).

## Supplementary Material

Supplementary Data
