# Supplementary material for: X-linked adrenoleukodystrophy: very long-chain fatty acid metabolism is severely impaired in monocytes but not in lymphocytes
Source: Hum Mol Genet. 2013 Dec 20;23(10):2542–50. doi: 10.1093/hmg/ddt645 (PMC3990157; doi:10.1093/hmg/ddt645)
Supplement: Supplementary Data [file supp_23_10_2542__index.html]

X-linked adrenoleukodystrophy: very long-chain fatty acid metabolism is severely impaired in monocytes but not in lymphocytes — X-linked adrenoleukodystrophy: very long-chain fatty acid metabolism is severely impaired in monocytes but not in lymphocytes — Supplementary Data 

# X-linked adrenoleukodystrophy: very long-chain fatty acid metabolism is severely impaired in monocytes but not in lymphocytes

## Supplementary Data

Supplementary Data

**Files in this Data Supplement:**

- Supplementary Data - pdf file
