# Supplementary material for: X-linked adrenoleukodystrophy: very long-chain fatty acid metabolism is severely impaired in monocytes but not in lymphocytes
Source: Hum Mol Genet. 2013 Dec 20;23(10):2542–50. doi: 10.1093/hmg/ddt645 (PMC3990157; doi:10.1093/hmg/ddt645)
Supplement: Supplementary Data [file supp_ddt645_ddt645supp.pdf]

SUPPLEMENTARY DATA

Supplementary Table 1: White blood cell count and C-reactive protein of AMN patients.

ID	Age (yrs)	Leucocytes (4-10 G/l)[a]	Band neutrophils (< 0.5 G/l)	Neutrophils (2-7.5 G/l)	Eosinophils (< 0.4 G/l)	Basophils (<0.4 G/l)	Monocytes (< 1.2 G/l)	Lymphocytes (1-4 G/l)	CRP (< 0.5 mg/dl)
P1	40	4.9	0.2	2.5	0.2	0.0	0.4	1.5	0.1
P2	37	5.8	0.2	2.4	0.3	0.0	0.4	2.5	-
P3	29	5.3	0.3	2.4	0.2	0.0	0.4	2.0	0.1
P4	43	10.7	0.4	6.4	0.2	0.0	0.5	3.1	0.1
P5	39	6.6	0.3	3.3	0.1	0.0	0.4	2.5	0.1

[a]Reference values are indicated for each cell population as giga pro litre (G/l) or mg/dl.

Supplementary Table 2: Specific magnetic antibodies (MACS® beads) used.

Cell type	*Specificity*	*Isotype*	*Type of selection*	*Product No.* (Miltenyi Biotech)
Pan T cells	CD3	Mouse IgG2a	positive	130-050-101
T helper cells	CD4	Mouse IgG1	positive	130-045-101
Cytotoxic T cells	CD8	Mouse IgG2a	positive	130-045-201
NKT cells	CD56	Mouse IgG1	negative	130-093-064
Monocytes	CD14	Mouse IgG2a	positive	130-050-201
Neutrophils	CD16	Mouse IgM	positive	130-045-701
B cells	CD19	Mouse IgG1	positive	130-050-301
NK cells	CD56	Mouse IgG1	positive	130-050-401

Supplementary Table 3: Thermocycler programme for qRT-PCR.

Gene	Initial denaturation		Cycles	Denaturation		Annealing/Elongation	
	Temperature	Time		Temperature	Time	Temperature	Time
ABCD1	95 °C	30 s	50	95 °C	5 s	58 °C	10 s
ABCD2	95 °C	30 s	50	95 °C	5 s	60 °C	10 s
ABCD3	95 °C	30 s	50	95 °C	5 s	60 °C	5 s
HPRT	95 °C	30 s	50	95 °C	5 s	57 °C	5 s
GAPDH	95 °C	30 s	50	95 °C	5 s	56 °C	10 s

Supplementary Figure S1

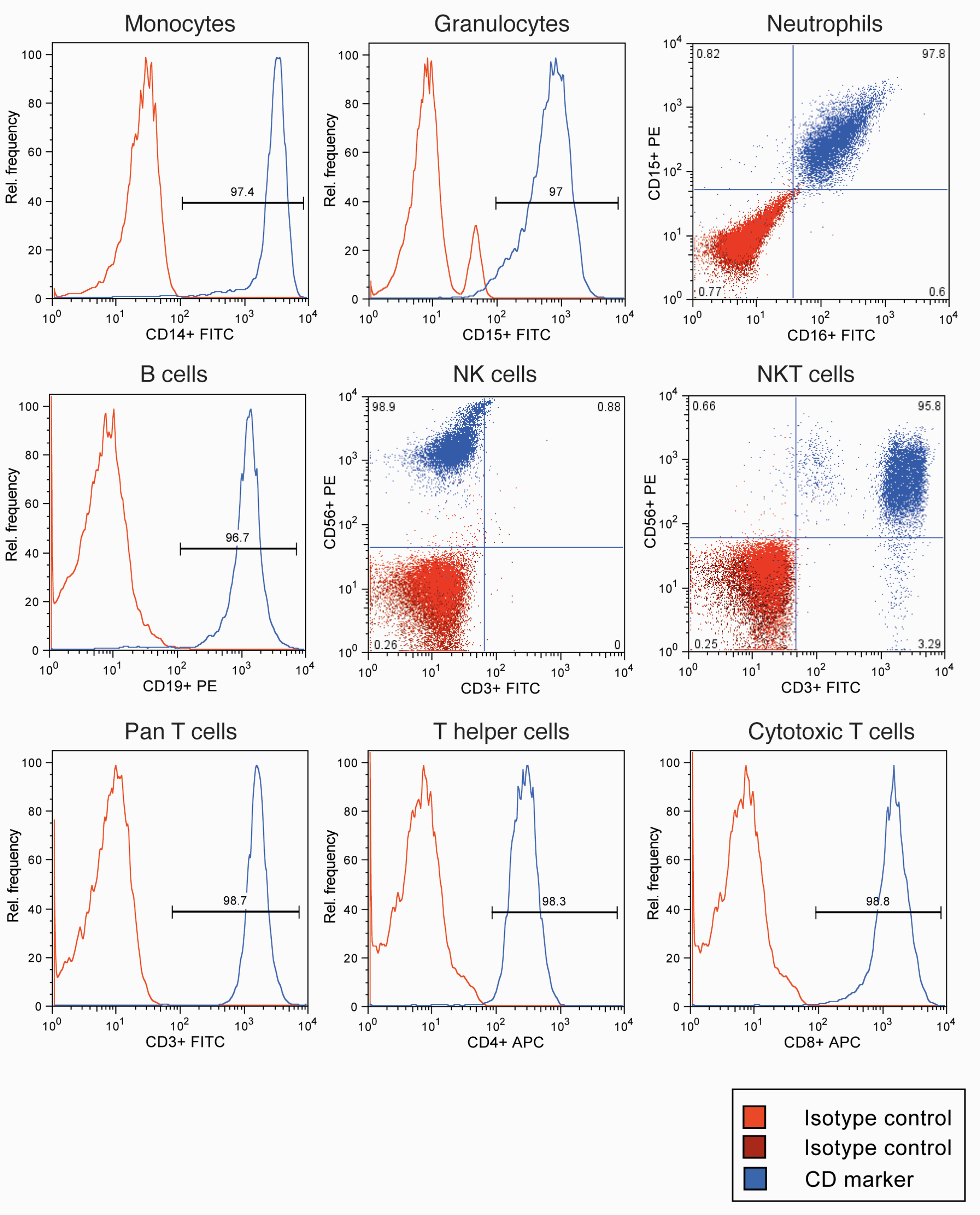

Supplementary Figure S1: Purity of isolated immune cells by flow cytometry. Representative analyses of cell purity for: monocytes ($CD14^+$) 97.4%; granulocytes ($CD15^+$) 97%; double-positive neutrophils ($CD15^+CD16^+$) 97.8%; B cells ($CD19^+$) 96.7%; single-positive NK cells ($CD56^+$) 98.9%; double-positive NKT cells ($CD3^+CD56^+$) 95.8%; Pan T cells ($CD3^+$) 98.7%; T helper cells ($CD4^+$) 96.3% and T cytotoxic cells ($CD8^+$) 96.8%. Isotype controls are shown in red (light and dark) and cell specific CD markers in blue. Relative frequency of each maximum cell count is indicated on the y-axis of the histograms. The dot plots show double stainings with fluorescence intensity for the PE channel on the x-axis and the FITC channel on the y-axis. Each dot represents a cell count.

Supplementary Figure S2

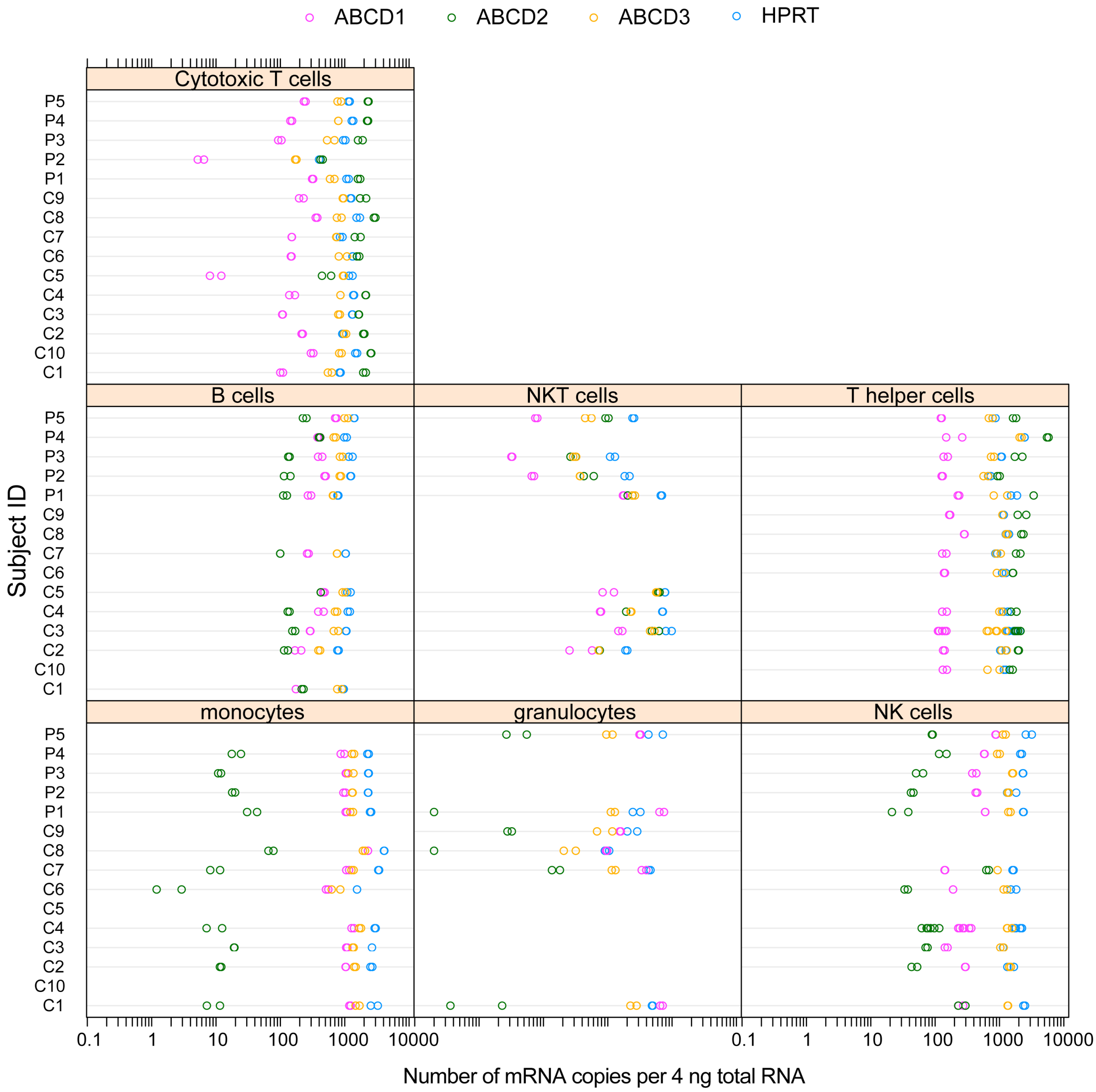

Supplementary Figure S2: Raw data of qRT-PCR analyses. mRNA copy number per 4 ng total RNA of ABCD1, ABCD2, ABCD3 and HPRT in different immune cells of AMN (P) and control (C) subjects are indicated on a log scale.

Supplementary Figure S3

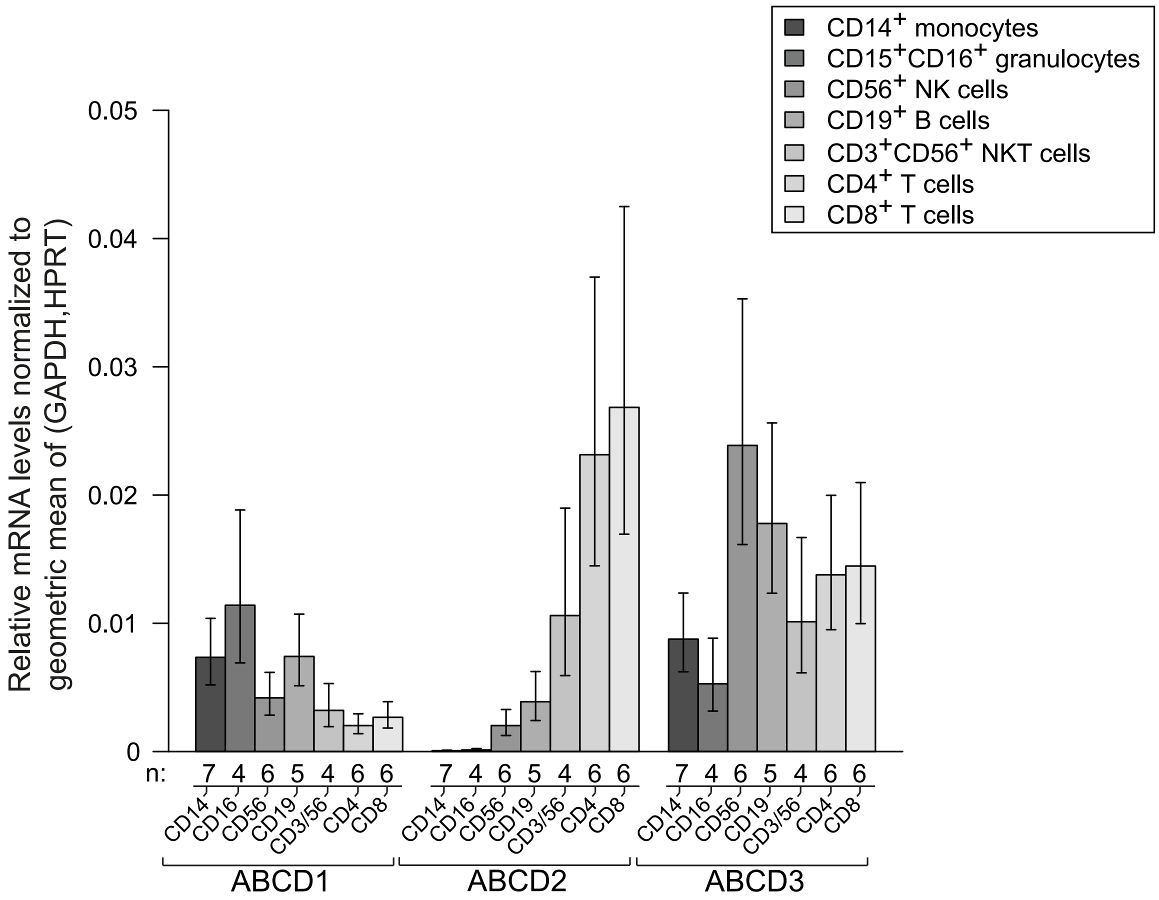

Supplementary Figure S3: Peroxisomal ABCD transporters are differentially expressed in the main immune cells. The mRNA levels of ABCD1, ABCD2 and ABCD3 were measured by qRT-PCR in the indicated immune cell types in healthy controls. Absolute copy numbers of the target genes were normalised to the geometric mean of the reference genes. Values represent means ± SEM. The number of individuals (*n*) is indicated below the graphs.

Supplementary Figure S4

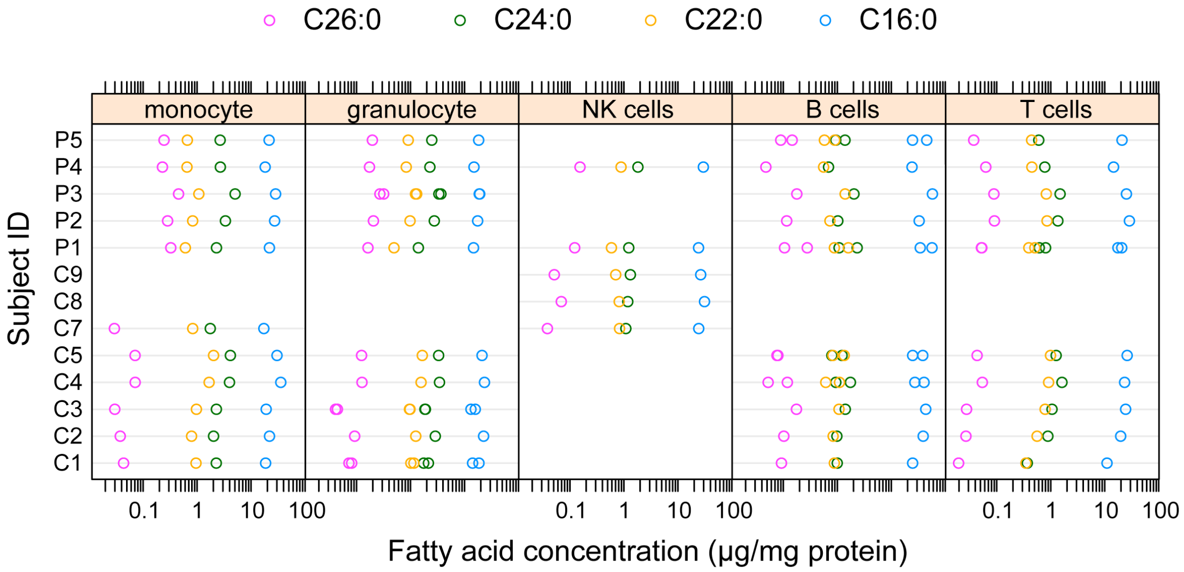

Supplementary Figure S4: Raw data of fatty acid measurements by GC-MS. The concentrations of C26:0, C24:0, C22:0 and C16:0 in different immune cell types of AMN (P) and control (C) subjects are expressed as µg/mg cellular protein on a log scale.

Supplementary Figure S5

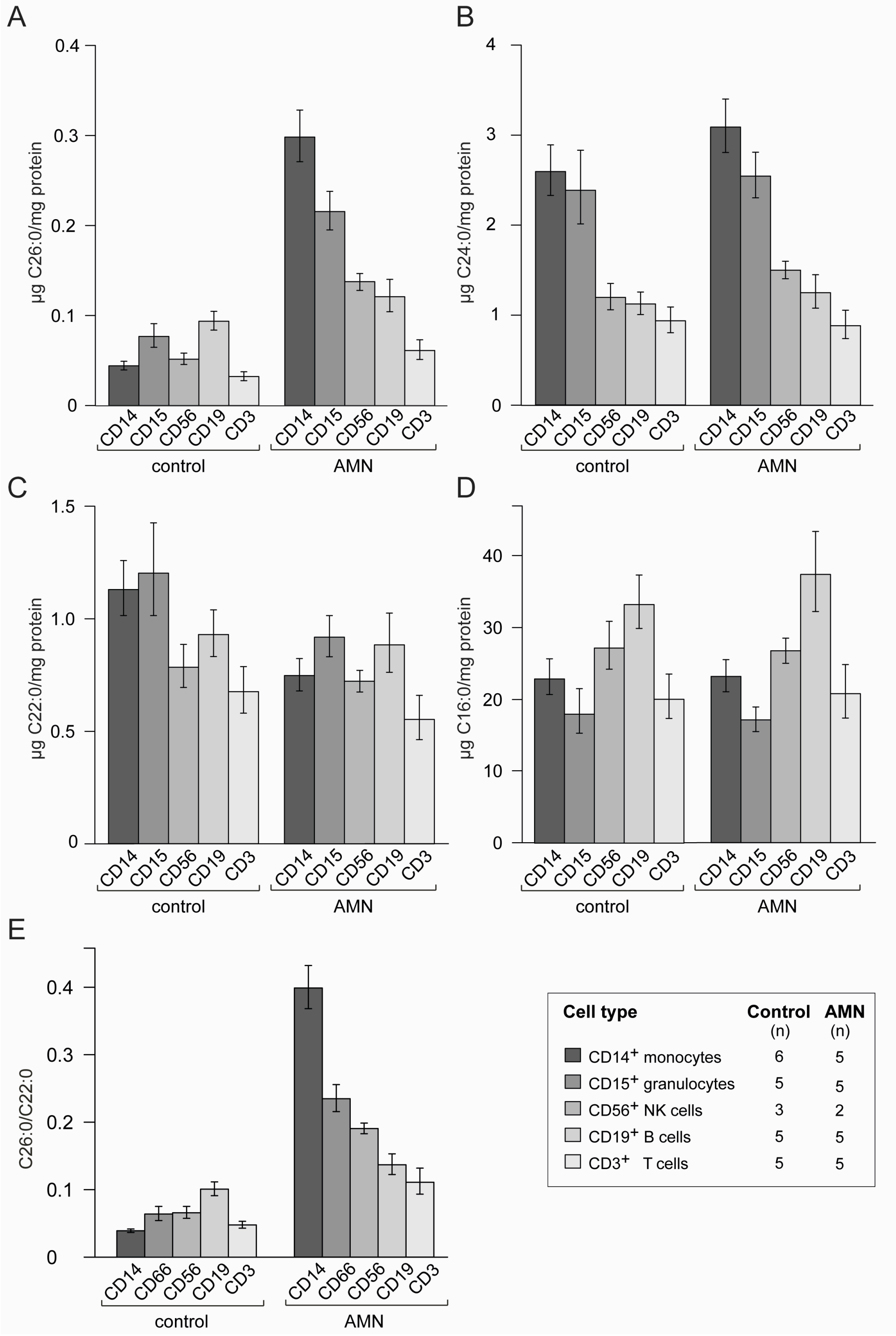

Supplementary Figure S5: Absolute amounts of fatty acids and C26:0/C22:0 ratio in immune cells of healthy controls and AMN patients. The concentrations of C26:0, C24:0, C22:0 and C16:0 were determined by GC–MS in monocytes ($CD14^+$), granulocytes ($CD15^+$), NK cells ($CD56^+$), B cells ($CD19^+$) and T cells ($CD3^+$). The absolute amounts (µg/mg protein) of **(A)** C26:0, **(B)** C24:0, **(C)** C22:0, **(D)** C16:0 and **(E)** the relative level of C26:0 expressed as ratio to C22:0 are shown for AMN and healthy controls. Values represent means ± SEM. For all panels, the number of individuals (*n*) is indicated in the inset of E. Note the different scaling.

Supplementary Figure S6

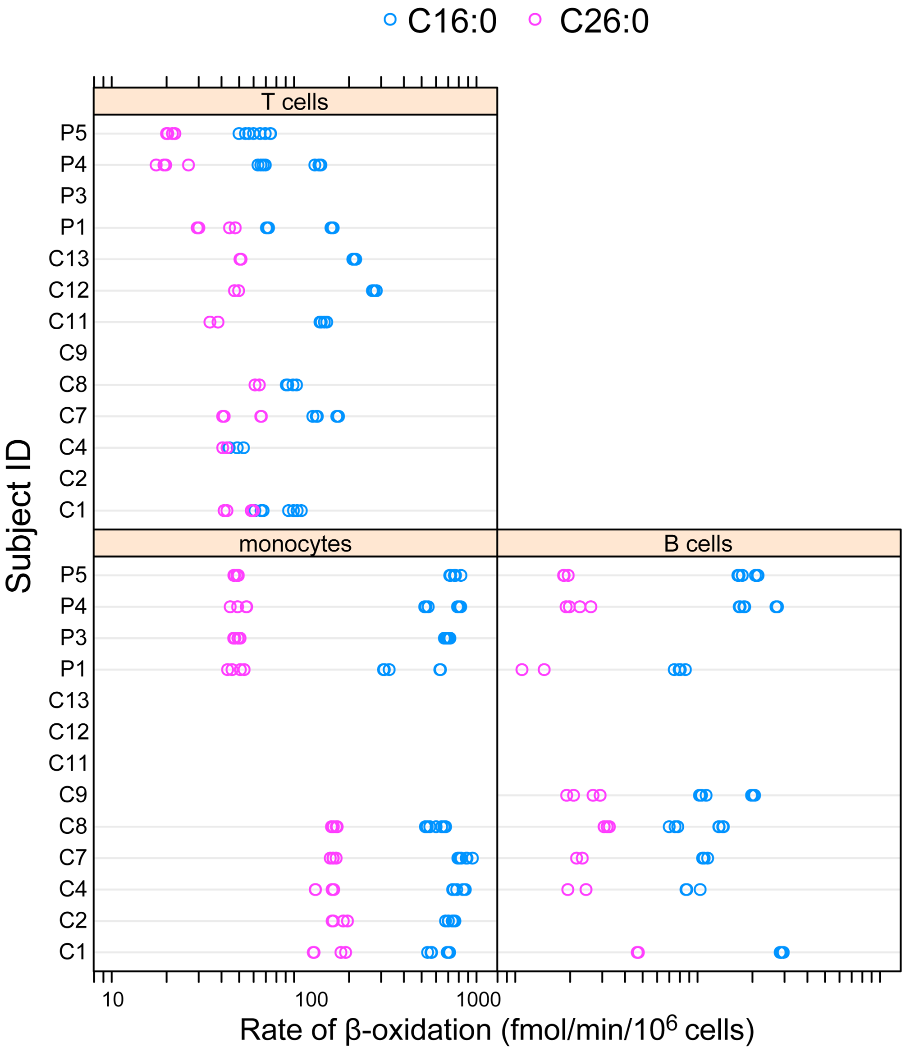

Supplementary Figure S6: Raw data of β-Oxidation activity in immune cells of healthy controls and AMN patients. The activity of **(A)** peroxisomal C26:0 and **(B)** mitochondrial C16:0 β-oxidation were measured in monocytes ($CD14^+$), B cells ($CD19^+$) and T cells ($CD3^+$). The rate of β-oxidation is expressed as fmol labelled acetate released/min/10^6 cells on a log scale.
