# Supplementary material for: X-linked adrenoleukodystrophy: very long-chain fatty acid metabolism is severely impaired in monocytes but not in lymphocytes
Source: Hum Mol Genet. 2013 Dec 20;23(10):2542–50. doi: 10.1093/hmg/ddt645 (PMC3990157; doi:10.1093/hmg/ddt645)
Supplement: Supplementary Data [file supp_ddt645_ddt645supp.doc]

**SUPPLEMENTARY DATA**

Supplementary Table 1: White blood cell count and C-reactive protein of AMN patients.

| ID | Age  (yrs) | Leucocytes  (4-10 G/l)^a^ | Band neutrophils  (< 0.5 G/l) | Neutrophils  (2-7.5 G/l) | Eosinophils  (< 0.4 G/l) | Basophils  (<0.4 G/l) | Monocytes  (< 1.2 G/l) | Lymphocytes  (1-4 G/l) | CRP  (< 0.5 mg/dl) |
| --- | --- | --- | --- | --- | --- | --- | --- | --- | --- |
| P1 | 40 | 4.9 | 0.2 | 2.5 | 0.2 | 0.0 | 0.4 | 1.5 | 0.1 |
| P2 | 37 | 5.8 | 0.2 | 2.4 | 0.3 | 0.0 | 0.4 | 2.5 | - |
| P3 | 29 | 5.3 | 0.3 | 2.4 | 0.2 | 0.0 | 0.4 | 2.0 | 0.1 |
| P4 | 43 | 10.7 | 0.4 | 6.4 | 0.2 | 0.0 | 0.5 | 3.1 | 0.1 |
| P5 | 39 | 6.6 | 0.3 | 3.3 | 0.1 | 0.0 | 0.4 | 2.5 | 0.1 |

^a^Reference values are indicated for each cell population as giga pro litre (G/l) or mg/dl.

**Supplementary Table 2: Specific magnetic antibodies (MACS®** beads) used.

| *Cell type* | *Specificity* | *Isotype* | *Type of selection* | *Product No.*  (Miltenyi Biotech) |
| --- | --- | --- | --- | --- |
| Pan T cells | CD3 | Mouse IgG2a | positive | 130-050-101 |
| T helper cells | CD4 | Mouse IgG1 | positive | 130-045-101 |
| Cytotoxic T cells | CD8 | Mouse IgG2a | positive | 130-045-201 |
| NKT cells | CD56 | Mouse IgG1 | negative | 130-093-064 |
| Monocytes | CD14 | Mouse IgG2a | positive | 130-050-201 |
| Neutrophils | CD16 | Mouse IgM | positive | 130-045-701 |
| B cells | CD19 | Mouse IgG1 | positive | 130-050-301 |
| NK cells | CD56 | Mouse IgG1 | positive | 130-050-401 |

**Supplementary Table 3: Thermocycler programme for qRT-PCR.**

| Gene | Initial denaturation | | Cycles | Denaturation | | Annealing/Elongation | |
| --- | --- | --- | --- | --- | --- | --- | --- |
|  | Temperature | Time |  | Temperature | Time | Temperature | Time |
| ABCD1 | 95 °C | 30 s | 50 | 95 °C | 5 s | 58 °C | 10 s |
| ABCD2 | 95 °C | 30 s | 50 | 95 °C | 5 s | 60 °C | 10 s |
| ABCD3 | 95 °C | 30 s | 50 | 95 °C | 5 s | 60 °C | 5 s |
| HPRT | 95 °C | 30 s | 50 | 95 °C | 5 s | 57 °C | 5 s |
| GAPDH | 95 °C | 30 s | 50 | 95 °C | 5 s | 56 °C | 10 s |

**Supplementary Figure S1**


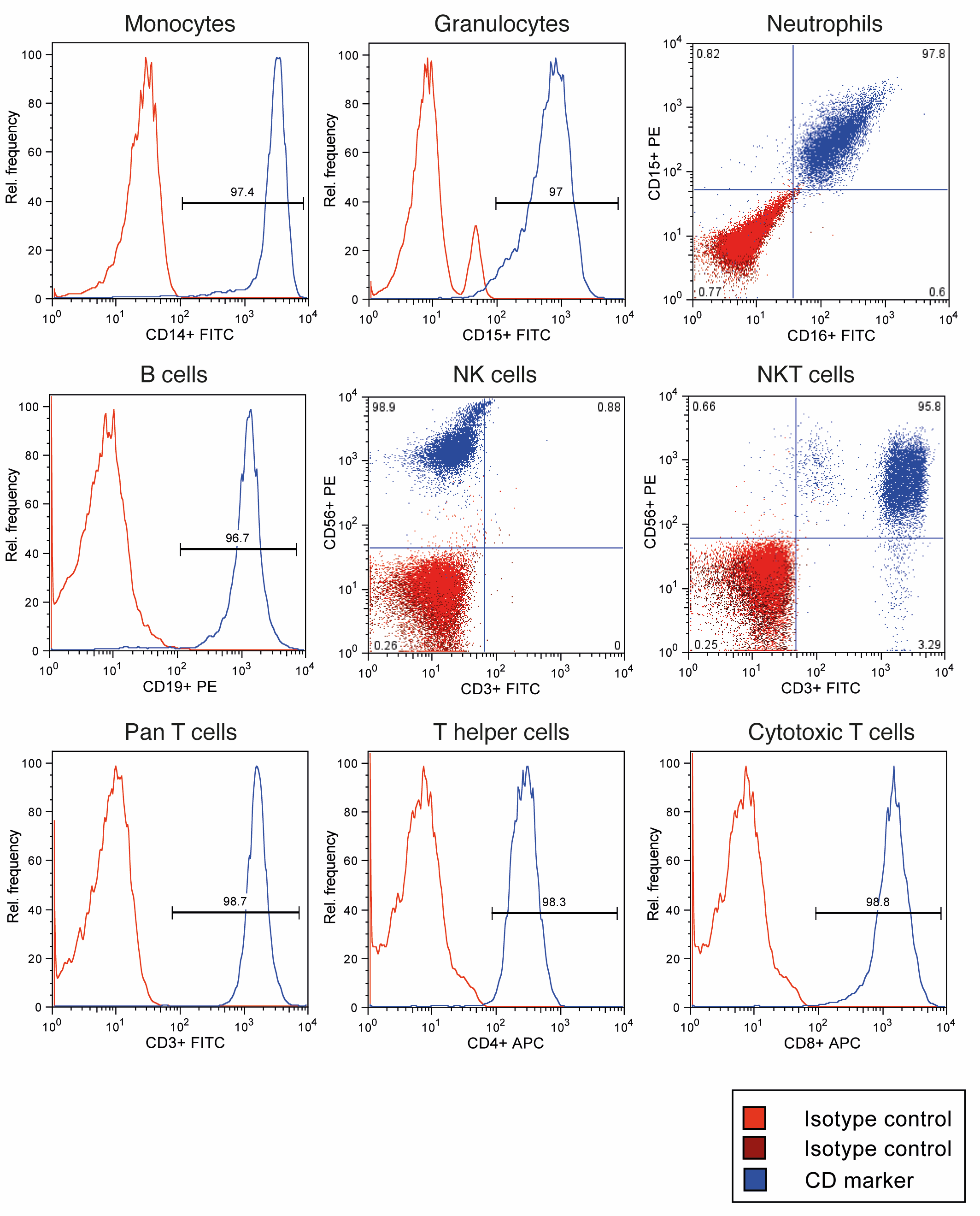


**Supplementary Figure S1: Purity of isolated immune cells by flow cytometry.** Representative analyses of cell purity for: monocytes (CD14^+^) 97.4%; granulocytes (CD15^+^) 97%; double-positive neutrophils (CD15^+^CD16^+^) 97.8%; B cells (CD19^+^) 96.7%; single-positive NK cells (CD56^+^) 98.9%; double-positive NKT cells (CD3^+^CD56^+^) 95.8%; Pan T cells (CD3^+^) 98.7%; T helper cells (CD4^+^) 96.3% and T cytotoxic cells (CD8^+^) 96.8%. Isotype controls are shown in red (light and dark) and cell specific CD markers in blue. Relative frequency of each maximum cell count is indicated on the y-axis of the histograms. The dot plots show double stainings with fluorescence intensity for the PE channel on the x-axis and the FITC channel on the y-axis. Each dot represents a cell count.

**Supplementary Figure S2**


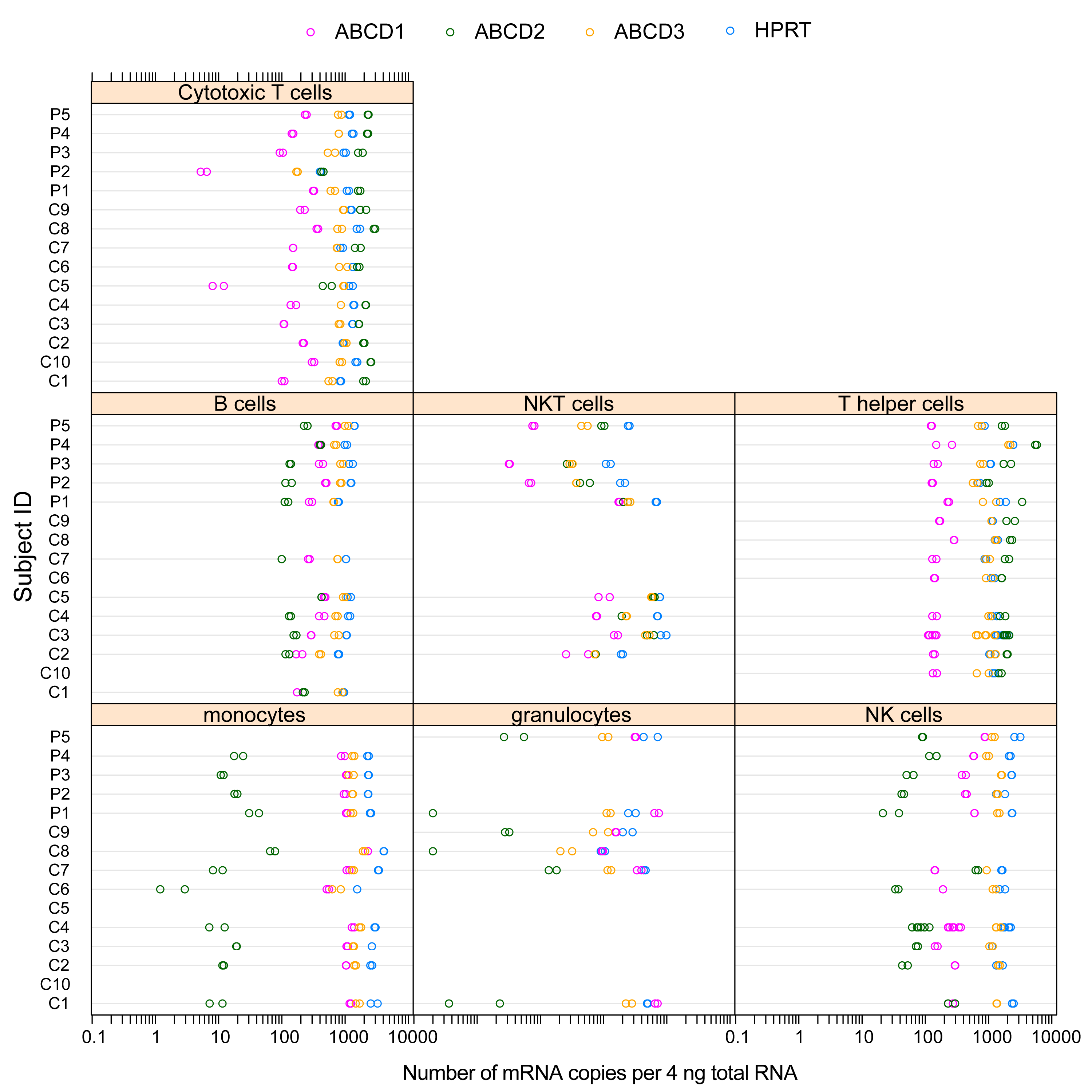


**Supplementary Figure S2:** **Raw data of qRT-PCR analyses.** mRNA copy number per 4 ng total RNA of ABCD1, ABCD2, ABCD3 and HPRT in different immune cells of AMN (P) and control (C) subjects are indicated on a log scale.

**Supplementary Figure S3**

**Supplementary Figure S3: Peroxisomal ABCD transporters are differentially expressed in the main immune cells.** The mRNA levels of ABCD1, ABCD2 and ABCD3 were measured by qRT-PCR in the indicated immune cell types in healthy controls. Absolute copy numbers of the target genes were normalised to the geometric mean of the reference genes. Values represent means ± SEM. The number of individuals (*n*) is indicated below the graphs.

**Supplementary Figure S4**


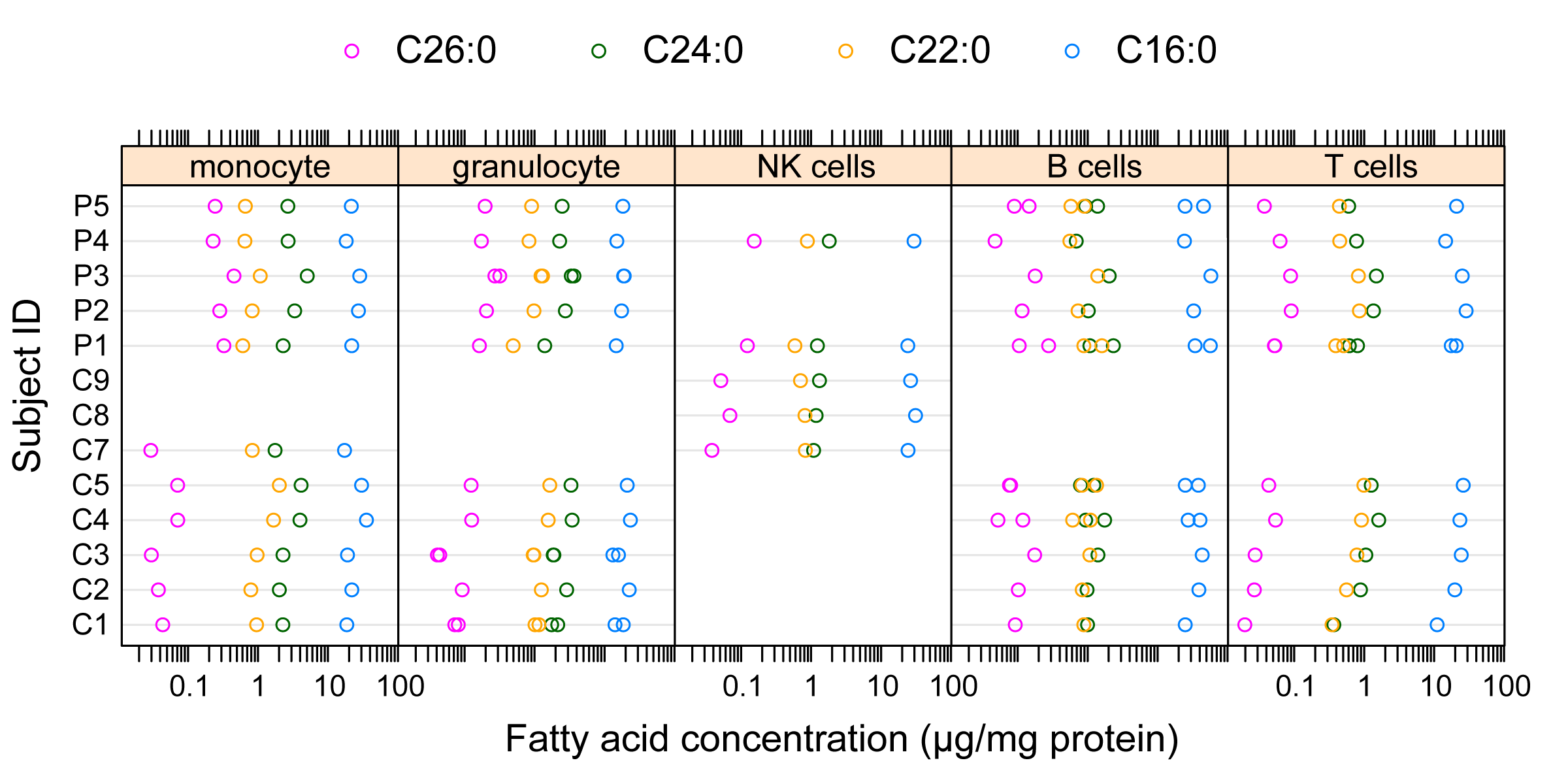


**Supplementary Figure S4:** **Raw data of fatty acid measurements by GC-MS.** The concentrations of C26:0, C24:0, C22:0 and C16:0 in different immune cell types of AMN (P) and control (C) subjects are expressed as µg/mg cellular protein on a log scale.

**Supplementary Figure S5**


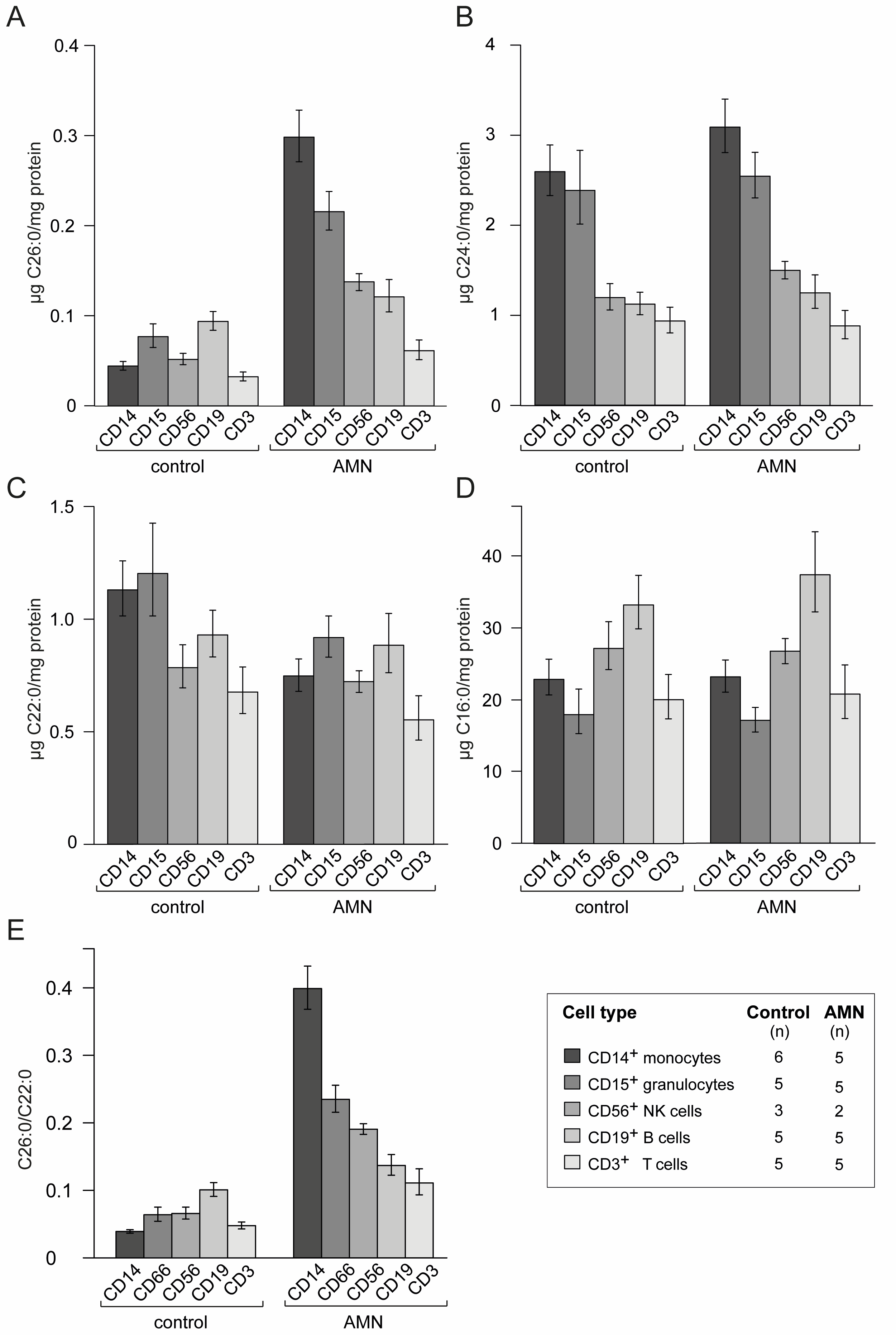


**Supplementary Figure S5: Absolute amounts of fatty acids and C26:0/C22:0 ratio in immune cells of healthy controls and AMN patients.** The concentrations of C26:0, C24:0, C22:0 and C16:0 were determined by GC–MS in monocytes (CD14^+^), granulocytes (CD15^+^), NK cells (CD56^+^), B cells (CD19^+^) and T cells (CD3^+^). The absolute amounts (µg/mg protein) of **(A)** C26:0, **(B)** C24:0, **(C)** C22:0, **(D)** C16:0 and **(E)** the relative level of C26:0 expressed as ratio to C22:0 are shown for AMN and healthy controls. Values represent means ± SEM. For all panels, the number of individuals (*n*) is indicated in the inset of E. Note the different scaling.

**Supplementary Figure S6**


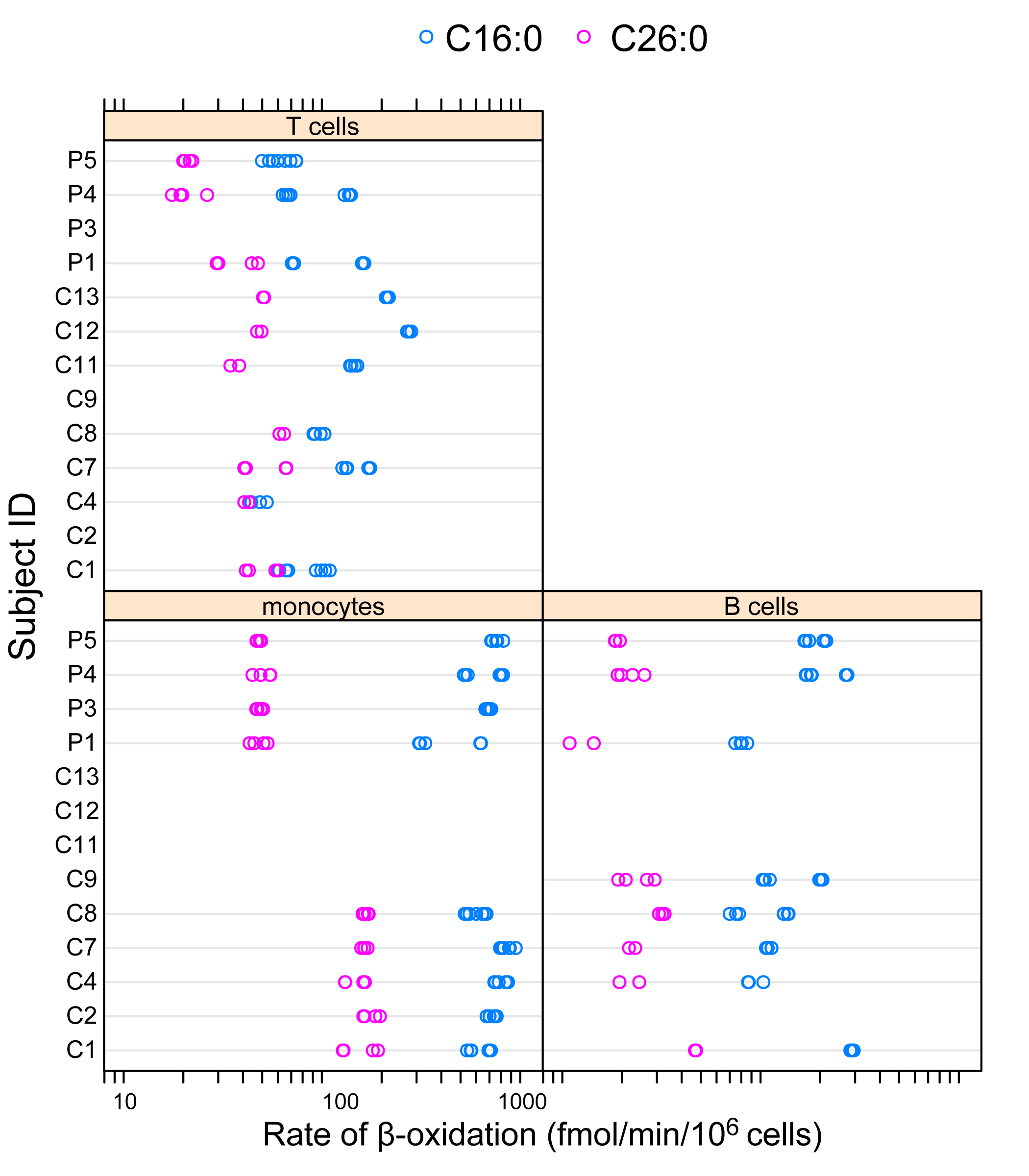


**Supplementary Figure S6: Raw data of β-Oxidation activity in immune cells of healthy controls and AMN patients.** The activity of **(A)** peroxisomal C26:0 and **(B)** mitochondrial C16:0 β-oxidation were measured in monocytes (CD14^+^), B cells (CD19^+^) and T cells (CD3^+^). The rate of β-oxidation is expressed as fmol labelled acetate released/min/10^6^ cells on a log scale.
